# Vegetarians: Past, Present, and Future Regarding Their Diet Quality and Nutritional Status

**DOI:** 10.3390/nu15163587

**Published:** 2023-08-16

**Authors:** Fanny Petermann-Rocha, Frederick K. Ho

**Affiliations:** 1 Centro de Investigación Biomédica, Facultad de Medicina, Universidad Diego Portales, Santiago 8370068, Chile; 2 School of Cardiovascular and Metabolic Health, University of Glasgow, Glasgow G12 8TA, UK; 3 School of Health and Wellbeing, University of Glasgow, Glasgow G12 8TB, UK; frederick.ho@glasgow.ac.uk

The term “vegetarian” usually refers to individuals who exclude meat, fish, poultry and/or their derived products from their diet. However, the label is used loosely, and different types of vegetarians exist. For instance, ovo-lacto-vegetarians (commonly referred as vegetarians) exclude meat, fish and their respective subproducts from their diet but eat milk and eggs, while vegans restrict all types of animal products, including milk, eggs, or any food product tested on animals. Pescatarians (vegetarians who eat fish), ovo-, or lacto-vegetarians are also types of vegetarianism that exist among the abovementioned groups. Irrespective of these types, vegetarian diets are often considered healthier alternatives to traditional diets as they do not contain meat and meat products. However, it should be noted that health benefits are not the sole reason people choose vegetarian diets. Other reasons include moral values, eating disorders, social interactions, personal identity, religious beliefs, cultural practices, and considerations of planetary health [1,2]. Given the variations and the multitude of underlying reasons, it is clear that vegetarian diets are heterogeneous and should not be defined based on the consumption of any type of meat.

Even though vegetarianism is proving increasingly popular among different age groups and populations across the globe, the concept is not new. In fact, one of the first available manuscripts regarding this topic was published in 1952 [3]. Around this time, researchers were particularly interested in “pure vegetarians”, considering the arteriosclerosis benefits of avoiding meat, eggs, and dairy products [4]. One of the first investigated populations was monks who followed ovo-lacto vegetarian diets due to their traditions. From this first investigation, it became evident that these individuals did not have weight or height deficits; in fact, they had a normal nutritional status [5,6]. For this reason, some previous studies associated the selection of “healthy foods” with vegetarians, likely due to the knowledge or awareness of how these diets benefit human health [7]. Nonetheless, since these first studies on monks, it became clearer that even when vegetarian diets could sufficiently provide most nutrients, they were also characterised by lower intakes of vitamin B12, calcium and omega-3, as well as iron, magnesium and zinc in some cases [5,8,9]. Vitamin B12, calcium, iron and omega-3 deficits are directly attributed to the lack of food rich in these nutrients. On the other hand, mineral deficiency is associated with a higher dietary fibre intake that can reduce the absorption and bioavailability of some minerals such as zinc, calcium, and iron [10,11].

Despite the lower level in several nutrients, adopting a vegetarian diet also offers various potential health advantages, such as reductions in blood pressure levels, triglycerides, cholesterol, glucose, and inflammatory markers [12,13,14]. Almost three decades ago, researchers proposed that vegetarians were less likely to be obese or develop some chronic conditions such as type 2 diabetes, hypertension, or cancer than those who followed traditional diets [15]. Moreover, since the 1990s, it has been highlighted that an adequate vegetarian diet can be beneficial for older adults if it is carefully planned to avoid nutrient deficiency [16,17]. However, these health benefits may vary according to the overall diet quality among vegetarians, which might be heterogeneous, as are their effects on nutritional status and health.

Since 2013, the role of vegetarian diets has been widely investigated in different epidemiological studies, which highlight that the risk of obesity, diabetes, cardiovascular disease, and cancer is lower in vegetarians than in non-vegetarians [18,19,20,21,22,23,24]. Clinical trials have shown that prescribing a vegetarian diet may reduce body weight [22,23]. Specifically, a meta-analysis highlighted that a vegetarian diet was associated with a mean weight change of 4.6 kg in a completer analysis [22]. Using data from the EPIC-Oxford study, researchers highlighted that vegetarians had lower body mass index (BMI), obesity, lower systolic blood pressure and hypertension than meat eaters. These differences were more significant in vegans than in lacto-ovo vegetarians. They also showed a lower risk of ischemic heart disease (risk ratio (RR): 0.68 [95% CI: 0.58 to 0.81]) and cancer at specific sites compared to meat eaters (for instance, RR_stomach_: 0.37 [95% CI: 0.10 to 0.69]; RR_bladder_: 0.62 [95% CI: 0.49 to 0.84]) [20]. A prospective UK study identified that vegetarians had a 32% (95% CI: 0.58, 0.81) lower risk of ischemic heart disease, as well as lower BMI and non-HDL concentrations [24]. Regarding lipid profiles, previous systematic reviews and meta-analyses also corroborated that, vegetarians may have lower plasma homocysteine and triacylglycerol concentrations compared with meat eaters [25,26]. The Seventh Day Adventists have also been extensively investigated since, due to their religion, they follow a vegetarian diet. Several studies have associated following this type of diet (either vegan, lacto-ovo or even semi-vegetarian) with a reduced risk of diabetes [12], all cancers combined [20], cardiovascular disease [20] and all-cause mortality [27]. Conversely, a study from the UK highlighted that pescatarians might have a lower risk of cardiovascular disease than vegetarians, likely because fish is an essential source of PUFA (mainly n-3), vitamin D and selenium, which are cardioprotective nutrients [18].

However, research findings are inconsistent, partly due to the different definitions of vegetarians [28]. On the one hand, the classic dichotomic classification of a vegetarian is an individual who does not eat meat (including chicken and fish). On the other hand, a plant-based diet is associated with several benefits [29] and is frequently synonymous with vegetarianism. Well-planned vegetarian or vegan diets can supply all nutrients required for a balanced lifestyle. However, even if people do not eat meat or its subproducts, they can still consume a large amount of unhealthy foods. For instance, in an Asian Indian cohort, Borude S. identified that vegetarian patterns are associated with a higher incidence of morbid obesity and increased the risk of undergoing bariatric surgery [30]. The author showed that Indian vegetarians are more likely to eat butter, ghee, snacks, and honey since they follow this type of diet, not due to health awareness but due to cultural traditions [30]. Moreover, Gehring et al. showed that both ovo-lacto-vegetarian and vegan diets are associated with a higher consumption of ultra-processed food. In fact, ultra-processed foods account for around 37.0% and 39.5% of the total energy intake of ovo-lacto-vegetarians and vegans, respectively [31]. Plant-based sausages or hamburgers can also contain high salt concentrations and levels of saturated fat.

Considering that the number of vegetarians has risen in the last few years, the food industry has also found a new economic opportunity. While vegetarian food was limited to specific or local stores in the past, today, several vegetarian brands and restaurants have changed their products for their target audience. One product that has gained popularity among vegetarians is cheese. According to a recent study published by Docherty and Jasper, vegetarians include cheese in many meals as a flavour enhancer in the absence of meat [32]. In this context, the healthy benefits associated with a vegetarian diet may be attributable to those who adhere to a plant-based diet, since individuals with unhealthy diet patterns also tend to have worse lifestyles overall [33].

Moreover, it is important to acknowledge the different nutritional qualities among vegetarian diets. For instance, Clarys et al. highlighted that vegans tended to have lower energy, protein, saturated fat, calcium intake, and higher dietary fibre intake than an omnivorous diet [34]. Hence, vegetarians may be at risk of inadequacy of vitamins and minerals that are essential to maintain an equilibrium in different body systems like bone [35,36] and blood health (anaemia [37,38]), homocysteine levels [39], sperm quality [40], mental health (associated with a higher depression risk [41]) and oral health (may have greater dental risk erosion [42]) as has been already highlighted [39,43].

Given the heightened awareness of the environmental impact of meat, it is likely that the number of vegetarians will continue to rise. However, a recent study showed that vegan diets were not necessarily associated with lower environmental footprints than ovo-lacto-vegetarian diets [44]. As summarised from the literature, adherence to a well-planned vegetarian diet with the characteristics of a plant-based diet may confer great overall health benefits. However, there remains concern regarding the limitations of vegetarian diets; they can be low in critical nutrients that play an essential role in human health. Since a vegetarian diet is not necessarily synonymous with a healthy diet, precautions must be taken when choosing a vegetarian diet, which must be carefully managed. Therefore, future studies are required for a better classification of vegetarianism, considering their consumption of other foods, such as ultra proceed foods.

All of the abovementioned aspects spark the following questions: Are vegetarians a homogeneous group? How do the nutritional status and diet quality of vegetarians compare to those of omnivores? Are there variations among different types of vegetarians? Do vegetarians have better well-being compared with people who have other types of diets? We hope these and other important questions can be answered in this Special Issue, which includes manuscripts focusing on nutrition, diet quality, dietary patterns, and/or well-being in observational and experimental studies carried out for all age groups. Ultimately, we hope the content will be useful for clinical practitioners and inspire further innovative research.
